# Central and peripheral haemodynamics at exercise onset: the role of central command

**DOI:** 10.1007/s00421-024-05513-3

**Published:** 2024-05-31

**Authors:** Gaia Giuriato, Stephen J. Ives, Cantor Tarperi, Lorenzo Bortolan, Federico Ruzzante, Antonio Cevese, Federico Schena, Massimo Venturelli

**Affiliations:** 1 Department of Neuroscience, Biomedicine and Movement Sciences, University of Verona, Verona, Italy; 2 Surgical, Medical and Dental Department of Morphological Sciences Related to Transplant, Oncology and Regenerative Medicine, University of Modena and Reggio Emilia, Modena, Italy; 3 Health and Human Physiological Sciences Department, Skidmore College, Saratoga Springs, NY USA; 4 Department of Internal Medicine, University of Utah, Salt Lake City, UT USA

**Keywords:** Blood flow, Hyperemia, Electrical stimulation, Voluntary contraction

## Abstract

**Purpose:**

The involvement of central command in central hemodynamic regulation during exercise is relatively well-known, although its contribution to peripheral hemodynamics at the onset of low-intensity contractions is debated. This study sought to examine central and peripheral hemodynamics during electrically-evoked muscle contractions (without central command) and voluntary muscle activity (with central command).

**Methods:**

Cyclic quadriceps isometric contractions (1 every second), either electrically-evoked (ES; 200 ms trains composed of 20 square waves) or performed voluntarily (VC), were executed by 10 healthy males (26 ± 3 years). In both trials, matched for force output, peripheral and central hemodynamics were analysed.

**Results:**

At exercise onset, both ES and VC exhibited equal peaks of femoral blood flow (1276 ± 849 *vs.* 1117 ± 632 ml/min, *p* > 0.05) and vascular conductance (15 ± 11 *vs.* 13 ± 7 ml/min/mmHg, *p* > 0.05), respectively. Similar peaks of heart rate (86 ± 16 bpm *vs.* 85 ± 16 bpm), stroke volume (100 ± 20 *vs.* 99 ± 27 ml), cardiac output (8.2 ± 2.5 *vs.* 8.5 ± 2.1 L/min), and mean arterial pressure (113 ± 13 *vs.* 113 ± 3 mmHg), were recorded (all, *p* > 0.05). After ~ 50 s, all the variables drifted to lower values. Collectively, the hemodynamics showed equal responses.

**Conclusion:**

These results suggest a similar pathway for the initial (first 40 s) increase in central and peripheral hemodynamics. The parallel responses may suggest an initial minimal central command involvement during the onset of low-intensity contractions, likely associated with a neural drive activation delay or threshold.

## Introduction

The regulation of skeletal muscle blood flow (BF) is a complex and multidimensional process. It involves the local integration of vasoconstrictive and vasodilatory stimuli to distribute BF toward active skeletal muscles (Joyner and Casey 2015), in order to match the supply of O_2_ to the metabolic demand during exercise. Multiple pathways converge to tightly regulate BF, with central command, autonomic responses, and peripheral phenomena emerging as pivotal regulatory mechanisms (Buckwalter and Clifford 1999; Stickland et al. 2011, 2007, 2008). Despite considerable advancements in our understanding, the response of BF during the transition from rest to exercise remains a subject of debate.

The origin of the cardiovascular responses at the onset and during exercise has received considerable attention (McCloskey and Mitchell 1972; Buckwalter and Clifford 1999; Hughson and Tschakovsky 1999; Delp 1999; Joyner and Proctor 1999). The literature on the contribution of central command to local regulation of BF in humans is heterogeneous (Ishii et al. 2013; Joyner and Dietz 2003). In one study, neural involvement in voluntary tasks was suggested, as seen in decreased popliteal artery blood flow during isometric ES (at 10% and 30% of MVC) compared to voluntary activity (Walker et al. 1988). On the contrary, subsequent research demonstrated similar responses in local circulation and vascular resistance during stimulated and voluntary intermittent calf contractions (Miller et al. 2000), contradicting earlier findings. Regarding the sympathetic mechanism underlying centrally induced vasodilation, two prevailing hypotheses emerge: sympathetic withdrawal and sympathetic cholinergic vasodilatation. Nevertheless, to date, the literature seems to lean towards denying the notion of centrally-induced vasodilator signaling to muscles in humans (Joyner and Casey 2015).

The alternative for the regulation of BF in skeletal muscle at the onset of exercise is the involvement of vasoactive factors released during muscle contraction. Indeed, the increase in shear stress at the onset of exercise permits the local release of vasodilators (e.g., nitric oxide, NO) that, coupled with alterations in the muscle metabolic milieu, play a crucial role in regulating BF (Ives et al. 1985; McCloskey and Mitchell 1972). These factors act to counterbalance the sympathetic-mediated vasoconstriction, leading to an enhancement in BF, a process also known as functional sympatholysis (Remensnyder et al. 1962). However, due to the complexity of the topic and/or specific experimental design employed, whether or not central command may transmit vasodilator signal to skeletal muscle at the onset of exercise, especially at low intensity, is still under debate.

Focusing on the central hemodynamics, during the initial phases of exercise the cardioacceleration is considered mainly modulated by the autonomic nervous system, and partially by afferent feedback (Fisher et al. 2015; Smith et al. 2006). Studies have revealed a neural basis for the chronotropic response and the maintenance of blood pressure (BP) during the onset of exercise (Marshall et al. 1961; Robinson et al. 1966). On the contrary, ES and voluntary contraction (VC) paradigms have revealed that the CO, HR, and ventilation responses were similar (Strange et al. 1993; Fisher et al. 2002). Specifically, it has been demonstrated an equal increase in central hemodynamics during electrically-evoked contractions and VC, in the first 15 s after the start of a 30% MVC exercise, suggesting a minimal role of central command in this response (Fisher et al. 2002; Fisher and White 2003).

 The controversial results in the literature stem from several task modalities (static or dynamic, rhythmic or sustained) and intensities (usually above the 10% MVC). However, the study by Ishii et al. (2013) reintroduced the idea that central command has a role in the human skeletal muscle blood flow control in a condition where supposedly muscle contraction is absent, like motor imagery. This suggests that central command may indeed be active at the onset of low-intensity muscle contractions. One way to study muscle contraction in humans is with voluntary exercise or muscle electrical stimulation, in particular during static (isometric) tasks. With electrical stimulation, the activation of central command is bypassed and muscle contraction can still be achieved in the absence of exercise. Considering this, at the onset of voluntary muscle contraction, the activation of central command is expected to induce hemodynamic changes (*i.e*., heightened heart rate, blood pressure, etc.), which would not occur during electrical stimulation. To test these changes, we compared central and peripheral hemodynamics during the onset of voluntary and electrically stimulated rhythmic low-intensity isometric quadriceps muscle contractions. With the assumption that ES would not evoke central command, we hypothesized that VC would exhibit greater central and peripheral circulatory responses, likely attributable to the influence of the central neural drive.

## Methods

### Participants

Young healthy males were recruited to participate in the study. Those with a history of cardiovascular, pulmonary, neurological, or metabolic disease or those taking regular medication were excluded. All participants provided written informed consent prior to any testing. The protocol was approved by the Institutional Review Board of the University of Verona and was conducted in accordance with the most recent revisions to the Declaration of Helsinki.

### Experimental design

**Fig. 1:**
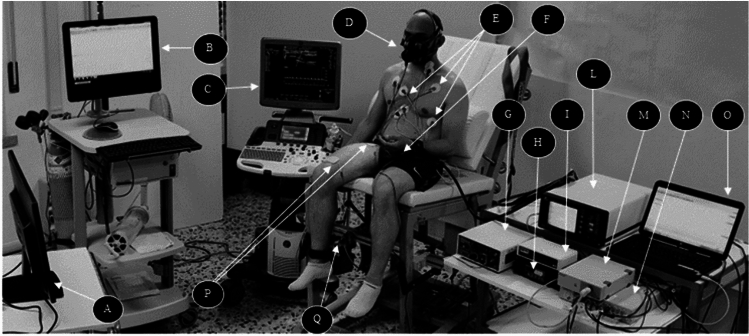
Representation of the set-up. **A** Monitor for visual feedback; **B** Metabolic cart; **C** Ultrasound Doppler; **D** Face mask; **E** ECG electrodes; **F** Pressure cuff; **G** Electrical stimulator; (**H**) EMG device; **I** ECG module (ADinstruments); **L** Plethysmography (Finapress device); **M** impedance electrocardiography (Physioflow); **N** Powerlab (AD Instruments); **O** Separate portable computer; **P** Electrical stimulation electrodes; **Q** Load cell

The participants were positioned on a comfortable reclining chair with their torso hip angle at 110°, and knees at 90° for the whole time of the study (Fig. 1). On the first day, the participants performed a maximal voluntary contraction (MVC) to compare the force expressed during the trials relative to individual maximal capacity, familiarized with the tasks, and the ES intensity was determined (Giuriato et al. 2020). The amplitude of the ES was determined with increments of 10 mA to the highest tolerable discomfort level. The discomfort level was measured on a visual analogue scale (VAS) scale from 0 (No pain) to 10 (Maximal pain), with a cut-off at level 4 (Tolerable). On the day of the test, participants rested seated for 10 min before data collection. They were asked to perform two different exercise tasks with the dominant leg: (1) 2 min of quadriceps ES at the frequency of 1 Hz (with the pre-determined stimulation intensity); (2) 2 min of VC of the leg extensors at the frequency of 1 Hz (auditive feedback with a metronome and a visual feedback of the generated force output were utilized to help the participants during the VC task). The two tasks were performed in a random order. The time between the two trials was 15 min and full recovery from the previous exercise trial was monitored with a metabolic cart (QuarkB^2^; COSMED, Rome, Italy). Full recovery was defined as oxygen uptake ($\dot{V}{\text{O}}_{2}$) reaching baseline values or less. FBF, respiratory exchanges, central hemodynamic and force output were recorded throughout the study (Fig. 1).

### Electrical stimulation

After proper preparation (shaving and cleaning), two stimulating electrodes with full-surface solid adhesive hydrogel were positioned on the quadriceps: one on the proximal part of the thigh and the second on the distal portion of the leg extensors, 3 cm above the patella. The discomfort level was measured on a Visual Analogue Scale (VAS) scale from 0 (No pain) to 10 (Maximal pain), with a cut-off at level 4 (Tolerable). The electrical stimulation was delivered as square waves using a constant current stimulator (Digitimer DS7h, Welwyn Garden City, UK), controlled by a customized program (Giuriato et al. 2020). An Arduino microcontroller was programmed to elicit trains of stimuli verified by a Tektronix 100 MHz oscilloscope. In detail, 20 square wave pulses of 260 ƞs were delivered to the muscle over a period of 200 ms, every second, for 120 s. To ensure similar forces during ES and VC, the force of VC was matched with the force produced during the max stimulation reached by the participant during the initial electrical ramp.

### Central hemodynamics

HR, SV and CO were collected using impedance electrocardiography (PhysioFlow, Ebersviller, FR) to provide continuous central hemodynamic measurements. Prior to the application of ECG electrodes, the skin was properly shaved and cleaned with alcohol. According to the standard location, brachial artery BP was measured manually at rest using a sphygmomanometer and a stethoscope's bell placed over the brachial artery just below the cuff's edge. Moreover, mean arterial pressure (MAP) was continuously recorded throughout the trials using a plethysmography device (Finapress^®^, FMS, Amsterdam, the Netherlands). Vascular conductance was therefore calculated using second-by-second recordings from the ultrasound and the Finapress as femoral blood flow x MAP^−1^.

*EMG.* To ensure a similar muscle mass activation during voluntary and electrically evoked contractions, the EMG activity of the vastus lateralis was recorded. After preparation (shaving, abrading and cleaning the skin with alcohol) two adhesive hydrogel electrodes were placed on the right vastus lateralis approximately 10 cm superior to the head of the fibula, 20 mm distance inter-electrode, in accordance with previously published procedures (8). The EMG signal was recorded by a wireless EMG apparatus (ZeroWire, Aurion, Italy). The EMG signal was amplified (× 100 with a differential amplifier) and sampled at 1500 Hz using the same A/D converter, PC and software described below.

### Femoral blood flow

BF of the exercising leg was measured by an experienced sonographer with a high-resolution duplex ultrasound Doppler (Logiq S7pro—General Electric Medical Systems, Milwaukee, WI). A 12–14 MHz probe was placed ~ 2–3 cm above the bifurcation of common femoral artery. Blood velocity (v) measurements were collected using a pulsed mode with the probe placed with an insonation angle of < 60 °, and the sample volume was centered and maximized to cover the vessel size. Measurements of the femoral artery diameter, from endothelium to endothelium, were obtained at rest in B-mode, with a 90 ° angle. The final value was averaged between three measurements taken during the systole, diastole, and the latent phase. FBF was automatically calculated by the Logiq S7pro device as follows:

$$FBF = v_{mean} \,\pi \,\left( {\frac{vessel\,diameter}{2}} \right)^{2} \,60$$

where FBF is in milliliters per minute.

### Pulmonary responses

Ventilation $\left( {\dot{V}_{{\text{E}}} } \right)$ and pulmonary gas exchange ($\dot{V}$O_2_,$\dot{V}$CO_2_) were measured breath-by-breath at rest and during the two trials using a metabolic cart (QuarkB^2^; COSMED, Rome, Italy).

*Torque.* Torque was collected using a strain gauge (System Pese, Milan, Italy; linear response 1500 N) connected with a non-compliant strap at the exercising limb and amplified and sampled at 1500 Hz using an external A/D converter. The live recording of the force was displayed on a monitor visible to the participants and thus, they were aware of the force outputs. Force output was measured throughout the two trials. In particular, the force output measured during the determination of the maximal level of tolerable ES was used as a target for the VC trial.

### Data collection and analysis

During each protocol, central and peripheral hemodynamic (HR, SV, CO, MAP, FBF, vascular conductance), torque and EMG were simultaneously collected using a commercially available data acquisition software (PowerLab; ML132/ML880; ADInstruments, Bella Vista, Australia) and recorded using LabChart v4.2 (ADInstruments, Bella Vista, Australia) on a separate computer. The EMG recordings were analyzed using the quantitative EMG analysis module in Labchart. The raw signal was smoothed using a triangular (Bartlett) window and the peaks detected were compared (%) to the MVC and normalized for the time.

Pulmonary responses were collected breath-by-breath using a metabolic cart (QuarkB^2^; COSMED, Rome, Italy). The blood velocity v_mean_ was collected with 1 Hz resolution on the Doppler ultrasound system for the first minute of rest, and then for 120 s during the tests. From the femoral artery diameter and the BF velocity, net FBF was calculated. Central hemodynamic data were collected using a PhysioFlow device for the first minute of rest and then for 120 s during the test. Absolute peak (peak), delta peak (Δpeak), and AUC (area under the curve) were calculated for each variable. The AUC (area under the curve) of each variable was integrated using the trapezoidal rule:

$$\int\limits_{a}^{b} {f(x)dx{\mkern 1mu} = {\mkern 1mu} \frac{{b - a}}{{2n}}} \left[ {y_{0} + 2y_{1} + 2y_{2} + \cdots + 2y_{{n - 1}} + y_{n} } \right]$$

### Statistical analysis

All statistics were performed on GraphPad Prism (V.6.0, GraphPad Software, San Diego, California USA). Data are presented as Means ± SD (ES *vs.* VC) throughout the paper and as Means ± SEM (ES *vs.* VC) in the figures, for visual clarity. A sample size of 10 participants was selected to ensure a statistical power higher than 0.80. Continuous measurements were analyzed using a two-way repeated measures ANOVA, while a two-tailed independent sample t-test was used to compare the AUCs and peaks. For all comparisons, statistical significance was declared when *p* < 0.05.

## Results

### Study participants

Ten young and physically active males participated in this study. The mean age of the participants was 26 ± 3 years, body weight 76 ± 8 kg, height 179 ± 6 cm and with a BMI of 21.2 ± 1.7 kg/m^2^.

*Baseline measurements.* The absolute resting levels of HR, SV, CO, MAP, FBF and vascular conductance were similar before the ES or the VC (*p* > 0.05). In accordance, the absolute resting levels of $\dot{V}_{{\text{E}}}$, $\dot{V}{\text{O}}_{2} ,\,\dot{V}{\text{CO}}_{2}$ were also similar before the ES or the VC (*p* > 0.05).

### Torque and EMG during evoked and voluntary contractions

**Fig. 2:**
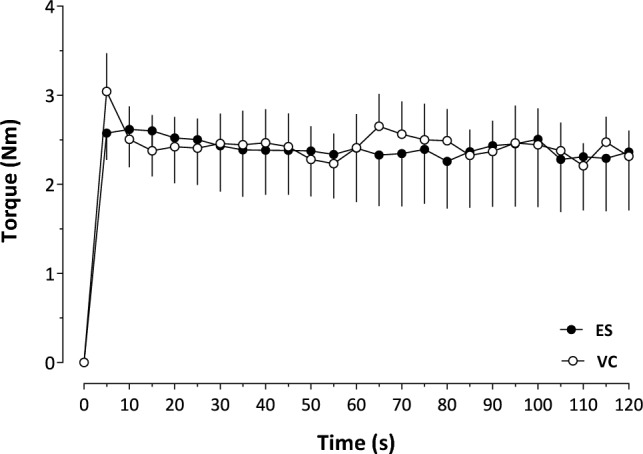
Profile of Torque over 120 s of electrical stimulation (ES) and voluntary contraction (VC) (*n* = 10). Values are presented as Means ± SEM

The torque evoked was approximately 5% of the individual’s voluntary maximal torque, with the EMG activity of the vastus lateralis being an average value of 1% of the MVC, as resembled with the study by Miller et al. (2000). The torque (Fig. 2) did not show a condition effect (*p* = 0.93), as well as an interaction effect between condition and time (*p* = 0.40). A time effect was yet observed (*p* < 0.001). The AUC of the torque expressed was similar (287 ± 75 *vs.* 291 ± 37 Nm s; *p* = 0.99) between the two conditions.

### Central hemodynamic during evoked and voluntary contractions

**Fig. 3:**
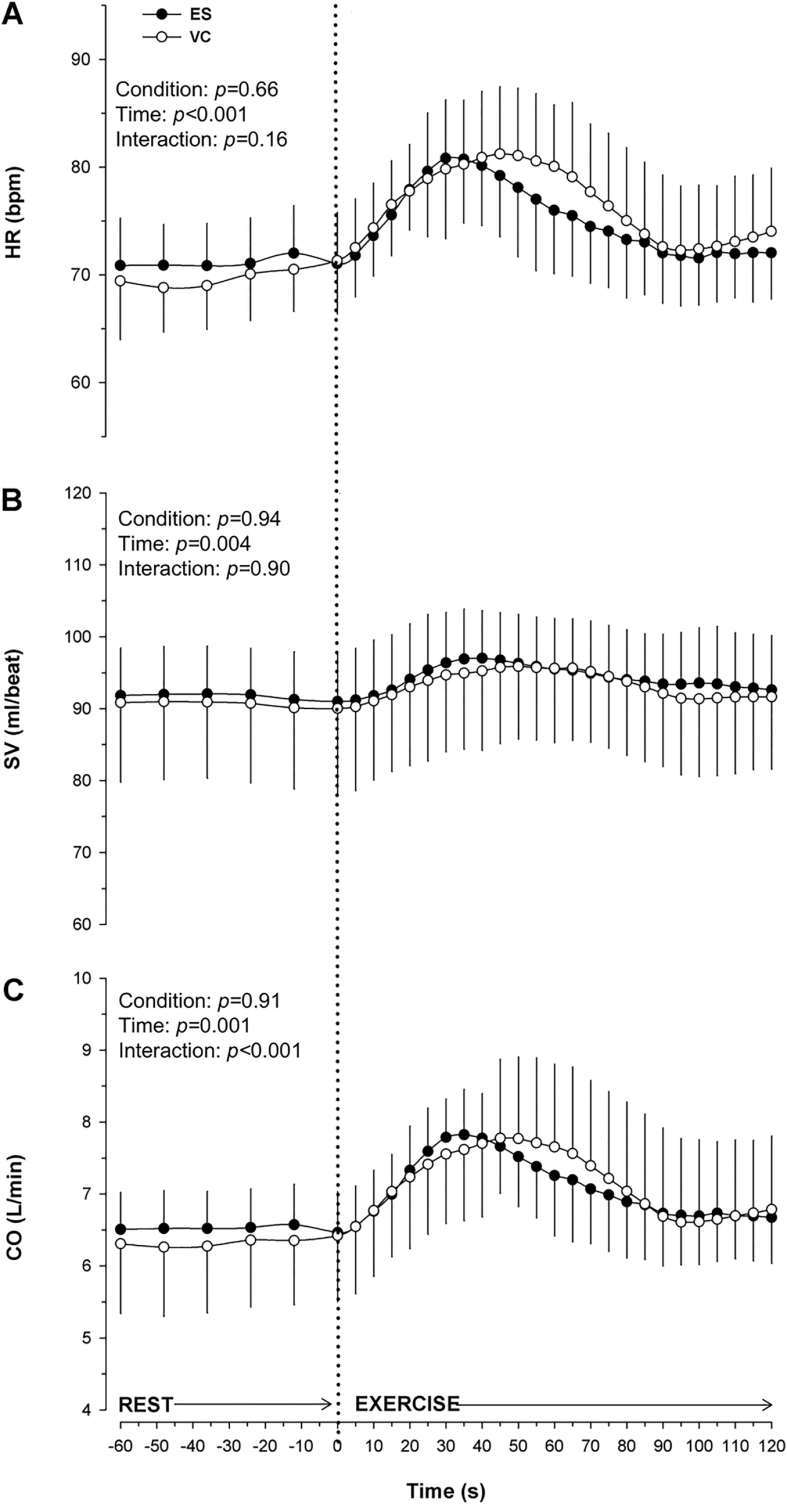
Central Haemodynamic responses over 120 s of electrical stimulation (ES) and voluntary contraction (VC). (**A**) *HR* heart rate, (**B**) *SV* stroke volume, (**C**) *CO* cardiac output. Values are presented as Means ± SEM

**Fig. 4:**
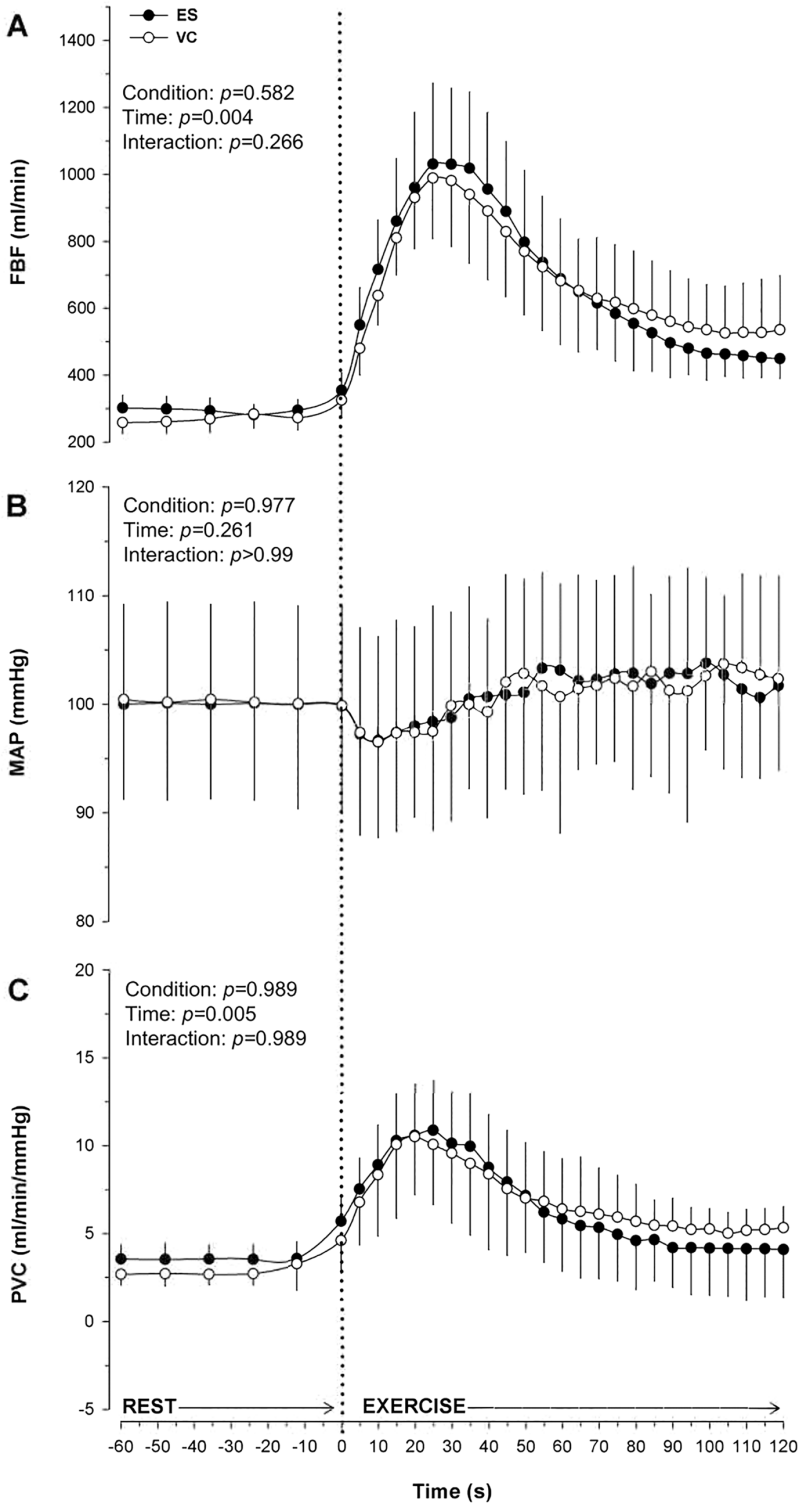
Peripheral cardiovascular regulation over 120 s of electrical stimulation (ES) and voluntary contraction (VC) (n = 10). A) Femoral blood flow (FBF); B) Mean arterial pressure (MAP); C) Vascular conductance. Values are presented as Means ± SEM

During the onset of the tasks, for approximately 40 s, HR, SV, and CO rapidly increased (Fig. 3). The CO showed a clear increase from the baseline to the peak of 35 ± 16% for ES and 48 ± 26% for VC (*p* = 0.18), the SV of 16 ± 6% and 16 ± 11% (*p* = 0.87), and the HR of 32 ± 12% and 32 ± 12% (*p* = 0.93), respectively (Table 1). The two conditions did not show differences in the AUC for all the parameters (Fig. 3; CO: *p* = 0.98; SV: *p* = 0.89; HR: *p* = 0.70). The HR, SV and CO demonstrated a significant time effect (HR: *p* < 0.001, SV: *p* = 0.004, CO: *p* = 0.001). However, no significant condition effect was observed for HR (*p* = 0.66), SV (*p* = 0.94), and CO (*p* = 0.91). Notably, there was no interaction effect for HR and SV (*p* = 0.16, *p* = 0.90, respectively), but an interaction effect was evident for CO (*p* < 0.001). The MAP showed no differences in the AUC (*p* = 0.87), and no interaction between condition and time, no main condition effect or time effect (all, *p* > 0.93; Fig. 4b).

**Table 1:** Central and peripheral hemodynamic responses during electrical stimulation (ES) and voluntary contraction (VC) of the quadriceps muscle in young healthy adults

	ES		VC	
	Peak	AUC	Peak	AUC
**HR, bpm**	86 ± 16	9183 ± 449	85 ± 16	8948 ± 423
**SV, ml**	100 ± 20	11,325 ± 599	99 ± 27	11,195 ± 770
**CO, L/min**	8.2 ± 2.5	859 ± 66	8.5 ± 2.1	861 ± 64
**FBF, ml/min**	1276 ± 849	81,778 ± 9307	1117 ± 632	84,280 ± 9611
**MAP, mmHg**	113 ± 13	12,007 ± 101	113 ± 3	11,988 ± 57
**PVC, ml/min/mmHg**	15 ± 11	955 ± 124	13 ± 7	960 ± 112

*HR* heart rate, *SV* stroke volume, *CO* cardiac output, *FBF* femoral blood flow, *MAP* mean arterial pressure, *PVC* peripheral vascular conductance. The mean of peaks (Peak) and area under the curve (AUC) data are presented as Means ± SD (*n* = 10). All, *p* > 0.05

### Peripheral hemodynamics during evoked and voluntary contractions

FBF shows a similar hyperemia at the onset of the exercise for the two conditions, with an increase in flow from baseline to the peak (reached in about 25 s) of 250 ± 152% during ES and of 216 ± 107% during VC (Table 1; *p* = 0.59). There was no significant condition effect or interaction between condition and time (Fig. 4a; all, *p* > 0.25). The blood flow AUC calculated during the conditions was similar (*p* = 0.65). Vascular conductance shows a rapid increase during both protocols with no differences in AUC (*p* = 0.99) and no interaction or condition effect (Fig. 4c; all, *p* > 0.98), suggesting a similar vasodilation.

### Pulmonary gas exchange during evoked and voluntary contractions

During the two trial $\dot{V}$O_2_ and $\dot{V}$CO_2_ showed a significant time effect (*p* < 0.001 and *p* = 0.036, respectively). However, here was no significant condition effect (all, *p* > 0.08) or interaction between condition and time (all, *p* > 0.16). The $\dot{V}_{{\text{E}}}$ did not show an effect of time, condition or an interaction between the two (all, *p* > 0.64). The peaks of $\dot{V}_{{\text{E}}}$ (17 ± 4 *vs.* 18 ± 5 L/min; *p* = 0.64), $\dot{V}{\text{O}}_{{2}}$ (594 ± 172 *vs.* 545 ± 127 ml/min; *p* = 0.49), and $\dot{V}{\text{CO}}_{{2}}$ (442 ± 103 *vs.* 463 ± 157 ml/min; *p* = 0.73), were similar during the two conditions.

## Discussion

**Fig. 5:**
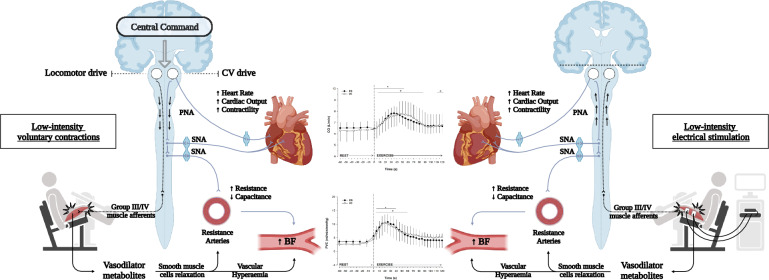
Central and peripheral Haemodynamic responses. At the onset of exercise, neural signals (i.e., central command) originating from the upper regions of the brain, are integrated with peripheral mechanisms (i.e., vasoactive factors). On the left side of the figure, the central command is active for the voluntary contraction, whereas on the right side the electrical stimulation is by-passing the central nervous system. The muscle contraction in the two conditions promotes a local release of vasoactive factors, that promote the relaxation of the resistance arteries with a consequent increase in blood flow (BF); however, the parallel peripheral vascular conductance (PVC) in the graph, suggests the minimal influence of the sympathetic nervous system (SNA) on the vasoconstriction. Moreover, the similar increase in cardiac output (CO) in the two conditions, reflects an equal involvement of the SNA and central command. Overall, during low-intensity muscle contractions, it is proposed that the cardiovascular drive (CV; grey lines) is delayed until the muscle needs further blood flow. Dotted lines: feedforward/feedback signals. Solid lines: feedforward signals

Central command is a mechanism contributing to locomotor and cardiovascular control during exercise. It involves the co-activation of cortical control mechanisms in the brain, which, along with feedback from mechano-receptors (sensing mechanical changes) and metabo-receptors (responding to metabolic byproducts) in the muscles, generates an integrated response to exercise (Amann et al. 2015). This study sought to parse the involvement of central command in the dynamics of peripheral and central circulation during the onset of low-intensity exercise in healthy humans. For this purpose, we compared the FBF and central hemodynamic changes during quadriceps rhythmic intermittent isometric voluntary and electrically-evoked contractions, matched for work. The findings of this study revealed similar hyperemia at the onset of low-intensity exercise in both conditions, likely mediated by local vasoactive factors released during the first 5–10 s of contractions. Similarly, the central hemodynamics (HR, SV, CO, MAP) during the onset of both trials were analogous. Collectively, during the onset of low-intensity contractions, the involvement of the central command in increasing the peripheral and central hemodynamic seems minimal (Fig. 5).

### The role of central command on peripheral haemodynamic

At the onset of muscular contractions, an increase in skeletal muscle vasodilatation occurs, with consequent hyperemia (Joyner and Casey 2015; Buckwalter and Clifford 1999; Remensnyder et al. 1962; Eldridge et al. 1985). Research indicates that the initial vasodilation is likely influenced by local factors, such as the mechanical deformation of vessels due to muscle contraction and an augmented release of local vasoactive substances (Joyner and Casey 2015; Saltin et al. 1998; Reed et al. 2000; Joyner and Proctor 1999). Conversely, studies exploring motor imagery propose that central command may transmit a vasodilator signal to bilateral skeletal muscles during both voluntary and imagined exercise (Ishii et al. 2013). However, investigations into the circulation of the contralateral limb suggest that hyperemia in this context may not be mediated by sympathetic activity (Fisher and White 2003). This highlights the complexity of blood flow regulation during exercise, where hyperemia can result from both mechanical stimuli and central command, with the involvement of sympathetic activity being variable and sometimes not evident.

The peripheral hyperemia observed in this study appears consistent across both voluntary low-intensity tasks and electrically stimulated (ES) force-matched contractions, where central command involvement is expected to be negligible. Similarly, studies involving paradigms where voluntary effort is absent, such as passive mobilization (Venturelli et al. 2014, 2017) or evoked contractions (Miller et al. 2000), indicate that the primary increase in muscular blood flow at exercise onset is likely mediated by local factors such as mechanical deformation or the muscle pump. Moreover, the similar transitory rise in vascular conductance in this study indicates a transient vasodilation of the vascular bed, likely due to local biochemical and mechanical factors.

It is important to note that the initial hyperemia likely depends on exercise modality, intensity and MAP (Joyner and Casey 2015). To avoid changes in MAP at the initial stages of exercise, studies found that very low-intensity dynamic exercise elicited a rapid shortening of R-R intervals at the onset of exercise (Nobrega et al. 1994), without major changes in perfusion pressure (Nobrega et al. 1994; Secher et al. 1988). Indeed, the peripheral hemodynamic responses observed in this study (Fig. 3) did not differ during voluntary and electrically-evoked contractions and are comparable to Miller et al. (2000).

### The role of central command on central haemodynamic

The autonomic nervous system plays an important role in generating a proportionate cardiovascular response during exercise. In particular, increases in HR and ventricular contractility during the onset of exercise result from the withdrawal of cardiac parasympathetic outflow, followed in later stages by a transient increase in sympathetic activity (Maciel et al. 1986; Fagraeus and Linnarsson 1976). The result we found in our low-intensity study showed that HR, SV and CO increased in a similar manner in the first 40 s after the onset of exercise during both VC and ES. However, after about 40 s from the onset of contractions, the HR and CO in the VC trial seem to be more sustained than in the ES trial. This result may represent a delayed activation of the central command in the regulation of HR and CO in the voluntary task (Rowell 2004). If we focus on just the first 40 s of exercise, our findings align with other studies conducted on healthy humans using ES (Kim et al. 1995; Strange et al. 1993; Fisher et al. 2002), suggesting a minimal involvement of central command in the regulation of central hemodynamics at the very beginning of low-intensity muscle contraction. This response aligns with a growing body of evidence indicating that other reflexes, like the exercise pressor reflex, are active and contribute to circulatory adjustments even during low-intensity exercise when oxygen delivery is considered sufficient (Amann et al. 2015; Grotle et al. 2020).

Similarly, a study conducted on cats following lateral spinal cord hemisection indicated that the cardio accelerator response to muscle contraction is not dependent on central command but is significantly influenced by the exercise pressor reflex (Murphy et al. 2013). However, this finding is not consistently supported in research involving anesthetized decerebrated cats and humans with paralyzed muscles (either due to pharmacological blockade or spinal cord injury). In these cases, the rapid cardiac acceleration at the onset of exercise is considered to be primarily mediated by the activation of cardiac sympathetic outflow rather than neural feedback from contracting muscles (Kadowaki et al. 2011; Secher 1985; Dela et al. 2003).

Overall, it seems that during the onset of voluntary low-intensity rhythmic intermittent isometric exercise in healthy humans, the increase in central hemodynamics is dual. At the very beginning is primarily caused by a withdrawal of cardiac parasympathetic outflow and feedback mechanisms from the contracting muscles, like the exercise pressor reflex. Later on, signals descending directly from the upper sites of central somatomotor areas (central command) increase the sympathetic tone.

## Limitations

This study is not without its limitations. Firstly, the absence of female participants limits the interpretation of the findings to a part of the population, as there may be sex-related differences (Bassareo and Crisafulli 2020; Macey et al. 2017). Secondly, the absence of a passive movement condition for comparative analysis, limits our ability to ascertain whether observed changes might be influenced by other variables (Shields et al. 2019). Thirdly, the task to follow a visual/auditory cue could represent a mental task typically accompanied by sympathetic stimulation and could have slightly influenced the hemodynamic responses (Burlingham et al. 2022). Future research incorporating a passive condition would provide a more robust basis for drawing definitive conclusions and better discerning the nuanced impacts studied here.

## Conclusion

This study aimed to understand the role and involvement of the central command in the central and peripheral hemodynamic changes during the onset of low-intensity rhythmic intermittent isometric exercise. To bypass the activation of the cardiovascular centers, electrically-evoked contractions were compared to voluntary contractions. We hypothesized a significant involvement of the central command in the regulation of peripheral and central circulation during the voluntary task. However, the current findings suggest a negligible role of central command on peripheral circulation, perhaps favoring processes mediated by local vasoactive factors released from the vascular endothelium or the exercise pressor reflex. Moreover, central hemodynamics appears not entirely governed by the autonomic response associated with the central command, but afferent feedback from contracting muscles likely plays a fundamental role at the very beginning of low-intensity contractions. However, after about 40 s, the rise in HR and CO in the voluntary contractions suggest an involvement of the central command. The similar responses observed within the initial 40 s of muscle contractions, alongside the delayed increase in HR and CO in the volunraty task, suggest a minimal central command involvement for low-intensity contractions, likely associated with a delay or threshold in neural drive activation (Fig. 5).

## Data Availability

The results of this study are presented clearly, honestly, and without fabrication, falsification, or inappropriate data manipulation.
